# Formulation of novel lipid-coated magnetic nanoparticles as the probe for in vivo imaging

**DOI:** 10.1186/1423-0127-16-86

**Published:** 2009-09-21

**Authors:** Huey-Chung Huang, Po-Yuan Chang, Karen Chang, Chao-Yu Chen, Chung-Wu Lin, Jyh-Horng Chen, Chung-Yuan Mou, Zee-Fen Chang, Fu-Hsiung Chang

**Affiliations:** 1 Institute of Biochemistry and Molecular Biology, College of Medicine, National Taiwan University, Taipei, Taiwan; 2 Department of Internal Medicine, National Taiwan University Hospital, Taipei, Taiwan; 3 Department of Life Science, College of Science and Engineering, Fu-Jen Catholic University, Taipei, Taiwan; 4 Department of Pathology, College of Medicine, National Taiwan University, Taipei, Taiwan; 5 Department of Electrical Engineering, National Taiwan University, Taipei, Taiwan; 6 Department of Chemistry, College of Science, National Taiwan University, Taipei, Taiwan

## Abstract

**Background:**

Application of superparamagnetic iron oxide nanoparticles (SPIOs) as the contrast agent has improved the quality of magnetic resonance (MR) imaging. Low efficiency of loading the commercially available iron oxide nanoparticles into cells and the cytotoxicity of previously formulated complexes limit their usage as the image probe. Here, we formulated new cationic lipid nanoparticles containing SPIOs feasible for *in vivo *imaging.

**Methods:**

Hydrophobic SPIOs were incorporated into cationic lipid 1,2-dioleoyl-3-(trimethylammonium) propane (DOTAP) and polyethylene-glycol-2000-1,2-distearyl-3-sn-phosphatidylethanolamine (PEG-DSPE) based micelles by self-assembly procedure to form lipid-coated SPIOs (L-SPIOs). Trace amount of Rhodamine-dioleoyl-phosphatidylethanolamine (Rhodamine-DOPE) was added as a fluorescent indicator. Particle size and zeta potential of L-SPIOs were determined by Dynamic Light Scattering (DLS) and Laser Doppler Velocimetry (LDV), respectively. HeLa, PC-3 and Neuro-2a cells were tested for loading efficiency and cytotoxicity of L-SPIOs using fluorescent microscopy, Prussian blue staining and flow cytometry. L-SPIO-loaded CT-26 cells were tested for *in vivo *MR imaging.

**Results:**

The novel formulation generates L-SPIOs particle with the average size of 46 nm. We showed efficient cellular uptake of these L-SPIOs with cationic surface charge into HeLa, PC-3 and Neuro-2a cells. The L-SPIO-loaded cells exhibited similar growth potential as compared to unloaded cells, and could be sorted by a magnet stand over ten-day duration. Furthermore, when SPIO-loaded CT-26 tumor cells were injected into Balb/c mice, the growth status of these tumor cells could be monitored using optical and MR images.

**Conclusion:**

We have developed a novel cationic lipid-based nanoparticle of SPIOs with high loading efficiency, low cytotoxicity and long-term imaging signals. The results suggested these newly formulated non-toxic lipid-coated magnetic nanoparticles as a versatile image probe for cell tracking.

## Background

Developing nanoprobe is important for diversely functional analysis in biomedical research [1]. Utilization of nanoparticles such as gold nanoparticles, quantum dots and superparamagnetic iron oxide nanoparticles (SPIOs) has been a fast growing area in molecular imaging. Nanosized gold particles are highly reactive in biological milieu, whereas quantum dots are nanocrystals of heavy metals in nature. But both of these nanoparticles are not biocompatible for human and for clinical practice. In this respect, SPIOs with polymeric coating, such as dextran, have been widely used in magnetic resonance (MR) imaging [2]. With its high spatial resolution, it has been widely used for tracking cell migration [3-5] and monitoring *in vivo *status of stem cell differentiation [6-9].

Because of the low efficiency of direct loading of these commercially available iron oxide nanoparticles into cells, transfection reagents such as cationic lipids, polylysine and protamine sulfate have been used for non-specific delivery of iron oxide nanoparticles into cells [10-13]. However, the formulated complexes are not stable and tend to aggregate in test tubes or even precipitate in the cell culture conditions, resulting in cytotoxicity [14]. Therefore, it remains a need to formulate new complexes without cytotoxicity for improving the delivery efficiencies to cells.

Polyethylene glycol (PEG)-modified lipids or polymers have been widely used to form the nanocarriers for pharmaceutical applications. Due to its small size, neutral surface charge and high hydrophilicity, PEG-modification greatly reduces the interactions of nanoparticles with plasma proteins and cells, ensuring their long circulation in the blood [15]. Similarly, charged phospholipids could, by themselves, form self-assembled film or particles in aqueous solutions. The diacyllipids, such as 1,2-dioleoyl-3-(trimethylammonium) propane (DOTAP) with strong positive charge and low transition temperature, may facilitate the quick formation of nanoparticles in physiological buffers [16,17]. Accordingly, we developed a novel formula of nanoparticles by combining PEG-lipid and DOTAP with SPIOs, and demonstrated the cellular uptake, biocompatibility and usage in the *in vivo *imaging of these new nanoparticles. The advantages of these new formulated lipid-coated SPIOs (L-SPIOs) include their small sizes, relative stability in physiological buffers, suitability for bimodal imaging and high loading efficiency to cells, allowing their usage as a versatile image probe for *in vivo *tracking analysis.

## Methods

### Materials

1,2-dioleoyl-3-(trimethylammonium) propane (DOTAP), polyethylene-glycol-2000-1,2-distearyl-3-sn-phosphatidylethanolamine (PEG-DSPE) and Rhodamine-dioleoyl-phosphatidylethanolamine (Rhodamine-DOPE) were obtained from Avanti Polar Lipids (Alabaster, AL, USA). These lipids were dissolved in chloroform individually, sealed in ampoules filled with argon gas and stored at -20°C before use. Dulbecco's Modified Eagle's Medium (DMEM) and RPMI-1640 medium were purchased from GIBCO™ Invitrogen Corporation (Grand Island, NY, USA). Other chemicals were purchased from Sigma-Aldrich (St. Louis, MO, USA).

### Cell cultures

Human cervical cancer cells (HeLa), human prostatic adenocarcinoma cell lines (PC-3), mouse neuroblastoma cells (Neuro-2a) and mouse colorectal adenocarcinoma cells (CT-26) were obtained from the American Type Culture Collections (ATCC) (MD, USA). HeLa, PC-3 and Neuro-2a cells were cultured in DMEM supplemented with 10% heat-inactivated fetal bovine serum (10% FBS-DMEM), whereas CT-26 cells were cultured in RPMI-1640 medium plus 10% serum. Routine cell culture was carried out at 37°C with supplementation of 5% CO_2_.

### Synthesis of hydrophobic SPIO nanoparticles

The hydrophobic SPIOs with size of 10 nm were synthesized using a seed-growth method by reducing Fe(acac)_3 _with 1,2-dodecanediol and protected by oleic acid and oleylamine in benzyl ether. After mixing in a round bottom flask and followed by heating to reflux (300°C) under nitrogen atmosphere for 1 h, the SPIOs were precipitated by adding ethanol, collected by centrifugation and dissolved in hexane in the presence of oleic acid and oleylamine. For lipid coating, the nanoparticles were precipitated and resuspended in chloroform before use.

### Preparation of lipid-coated SPIOs

Lipid-coated SPIOs (L-SPIOs) were prepared using a combination of the standard thin-film hydration method [18] and sonication process. Briefly, DOTAP and PEG-DSPE at 3:1 molar ratio in chloroform were mixed with hydrophobic SPIOs and trace amount of Rhodamine-DOPE, and then evaporated under reduced pressure. The resulted SPIO-lipid film was hydrated in distilled water to give the final concentrations of cationic lipids at 200 μM and iron oxide at 375 μg/ml. The solution of L-SPIOs was intensively sonicated at 60°C for 20 min. They were then sorted by the magnet, filter-sterilized through a Millex 0.22-μm-diameter filter and stored at 4°C in argon gas before use.

### Size and zeta potential determinations of lipid-coated SPIOs

The diameters and zeta potential of L-SPIOs were determined using a Zetasizer Nano-ZS90 (Malvern Instruments ZEN3590, Worcs, UK). The size was analyzed by Dynamic Light Scattering (DLS), and the zeta potential was calculated by Laser Doppler Velocimetry (LDV) at 25°C with samples suspended in de-ionized water. Prior to use, all glass and plastic wares were pre-washed with filtered water to minimize particulate contamination.

### Cellular loading of lipid-coated SPIOs

HeLa, PC-3 and Neuro-2a cells were seeded individually in each well of the 24-well plates (2-5 × 10^4 ^cells/well) overnight before L-SPIOs loading. Trace amount of Rhodamine-DOPE in the L-SPIOs was an indicator for monitoring the translocation of nanoparticles. After incubating with L-SPIOs (1-2 nmole/well, equivalent 1.875-3.75 μg SPIO) at different time points with or without the application of magnetic field, cells were washed with PBS. The high resolution fluorescent images were recorded using a fluorescent microscope (Leica DM IRB with the halogen lamp).

### Prussian blue staining

HeLa cells were seeded in a 24-well plate (5 × 10^4^/well) overnight and treated with L-SPIOs (1 nmole/well) for 2 h, with or without magnetic field. These cells were then washed three times with PBS and fixed in 4% formaldehyde solution for 30 min. After fixation, the cells were stained for the presence of intracellular iron with fresh prepared potassium ferrocyanate solution (mixture of equal volume of 4% potassium ferrocyanate with 4% hydrochloric acid) for 30 min. After wash three times with distilled water, the cells were then counterstained with Nuclear Fast Red Counterstain (Sigma-Aldrich) at room temperature for 5 min.

### Flow cytometry analysis of L-SPIO-loaded cells

Cells loaded with L-SPIOs were washed with PBS and trypsinized. Their cell loading efficiency was evaluated by scoring the percentage of Rhodamine fluorescent-positive cells using a FACS Calibur System (Becton-Dickinson, San Jose, CA, USA). The experiments were performed in triplicate, and 10,000 cells were counted in each experiment.

### Long-term growth and magnetic properties of L-SPIO-loaded cells

HeLa and PC-3 Cells were seeded in a 24-well plate (5 × 10^4^/well) overnight and treated with L-SPIOs (1 nmole/well) for 12 h. These cells were then washed with PBS twice and trypsinized. To analyze the effects of L-SPIO-loading on cell growth, both L-SPIO-loaded and control cells (1.5 × 10^5^/100-mm dish) were cultured for more than 10 days and cell numbers were counted at different time points by the hemocytometer. To determine the magnetic property of L-SPIO-loaded cells, cells were trypsinized at different time points and equal amount of cells (5 × 10^5^/500-μl medium in an Eppendorf tube) were sorted by the magnet stands and the magnet-bound cells were counted with a hemocytometer.

### Induction and assessment of neuron cell differentiation

For morphological differentiation analysis, the degree of neurite outgrowth was assessed by phase-contrast microscopy [19,20]. Briefly, Neuro-2a cells were treated with 5 mM retinoic acid in 1% FBS-containing medium for 60 h. The neurite-like processes longer than the major cell body diameter were defined as differentiated cells. In each well, two hundred cells in three random fields were counted. Data were expressed as percentage of neurite-bearing cells in total cell population.

### Imaging of tumor growth

For MR imaging of tumor growth, CT-26 cells (1 × 10^6^) were seeded in 100-mm dish overnight before L-SPIOs loading (20 nmole lipid/dish, equivalent to 37.5 μg SPIO per dish). After 16 h, CT-26 cells were removed and resuspended in PBS. Control and L-SPIO-loaded CT-26 cells (3 × 10^6^, respectively) were injected into the dermis tissue of Balb/c mice to initiate tumors. After 2 and 15 days, MR imaging were taken from the anesthetized animals. These experimental protocols were approved by National Taiwan University Institutional Animal Care and Use Committee. For MRI scanning of anesthetized animals, a 3T MR system (Biospec; Bruker, Ettlingen, Germany) was used. *T2*-weighted images were obtained by fast spin echo sequence, TR/TE of 3500/62 ms, sliced thickness = 1 mm, field of view = 40 × 20 mm^2^, matrix size = 256 × 256 and NEX = 5. The total scanning time was 4 min. T2*-weighted images were obtained by gradient echo sequence, TR/TE = 250/7 ms, sliced thickness = 1 mm, field of view = 40 × 20 mm^2^, matrix size = 256 × 256 and NEX = 10. The total scanning time was 8 min. After scanning at 15th day, the tumor nodules were excised, fixed in formalin, embedded in paraffin, and processed into 5-μm sections. They were then stained with haematoxylin and eosin (H&E) following the standard histological protocol.

## Results

### Characterization of lipid-coated SPIOs

**Figure 1:**
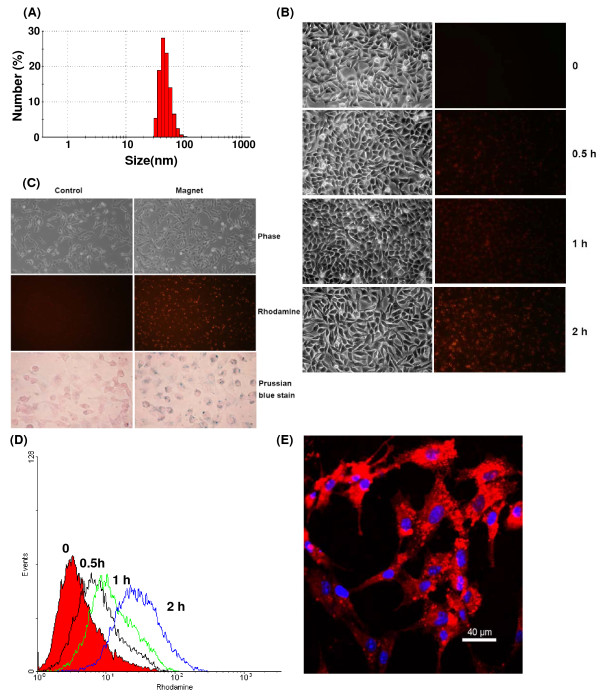
**Magnet effect on L-SPIOs loading in HeLa cells**. (A) Size distribution of L-SPIOs particles. (B) Images of HeLa cells treated with 1 nmole/well of L-SPIOs for 2 h in 10% FBS-DMEM with magnet (right panel) or without magnet (left panel). Trace amount of Rhodamine-DOPE was used as an indicator for localizing the position of the nanoparticles. Images were taken after L-SPIO treatment (phase and fluorescent images: 100× magnification; Prussian blue staining: 400× magnification). (C) Time-dependent effect of magnet on cell loading. HeLa cells were seeded overnight and treated with 1 nmole/well of L-SPIOs for 0, 0.5, 1 and 2 h. These fluorescent images were taken at different time points as indicated on the right. (D) Flowcytometric analysis of cells from (C) was shown. (E) Confocal image of L-SPIO-loaded HeLa cells. HeLa cells treated with L-SPIOs (1 nmole/well) for 16 h. After washed with cold PBS, cells were fixed and analyzed by a confocal microscope. Red fluorescence in cells was indicated as L-SPIOs, whereas blue fluorescence was nucleus. Scale bar = 40 μm. Data represent three independent experiments with similar results.

In pharmaceutical research, PEG-modified lipids have been widely used to form nanoparticles or micelles for drug targeting. Combined with a cationic lipid 1,2-dioleoyl-3-(trimethylammonium) propane (DOTAP), it could form a stable and uniform nanocarrier to encapsulate hydrophobic superparamagnetic iron oxide nanoparticles (SPIOs). The average size of these formulated lipid-coated SPIOs (L-SPIOs) was about 46 nm in diameter as shown in Fig 1A, carrying a positive zeta potential in deionized water (49.5 ± 0.8 mV). Incorporation of trace amount (less than 0.1%) of Rhodamine-DOPE as a fluorescent indicator did not affect the particle size. These L-SPIOs remained stable in a small and uniform size in distilled water or 5% glucose solution for more than three months. Culture medium, with or without serum, did not influence the size of L-SPIOs after magnetic sorting. (data not shown).

### Magnetic effect on the delivery of lipid-coated SPIOs into cultured cells

To facilitate the delivery of L-SPIOs into cells, a magnet apparatus was used in cell culture condition. HeLa cells (2-5 × 10^4^/well) were seeded and treated with 1 nmole/well L-SPIOs in 10% FBS-DMEM with or without the application of magnetic field. Trace amount of Rhodamine-DOPE was used as an indicator for localizing these nanoparticles. As shown in Figure 1 (Fig. 1B), the presence of magnetic field for 2 h remarkably improved the loading of L-SPIOs in HeLa cells without disrupting cell morphology. The magnetic field facilitated a time-dependent sedimentation and translocation of L-SPIOs into cells (Fig. 1C and 1D). The loading efficiency with magnet for 2 h was almost equal to that without magnet for 12 h. The loaded nanoparticles were mostly located in the cytoplasm as shown in the confocal microscopy, with only negligible L-SPIOs remaining bound to the cell membrane after 16 h of the nanoparticle treatment (Fig. 1E).

### Long-term viability and magnetic properties of L-SPIO-loaded cells

**Figure 2:**
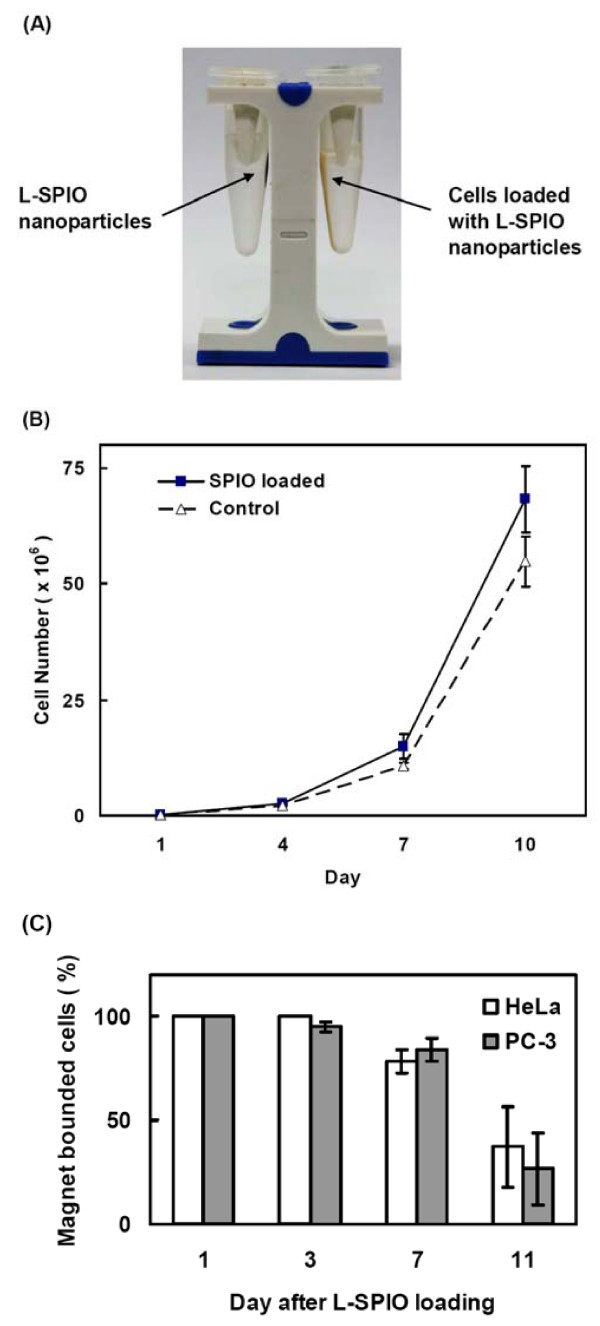
**Magnet effect on L-SPIOs and L-SPIO-loaded cancer cells**. (A) L-SPIOs in water were placed on the magnetic stand (left); HeLa cells treated with L-SPIOs (1 nmole/ml) for 12 h were on the right. Arrows indicate the position of L-SPIO nanoparticles or the L-SPIO-loaded cells. (B) Long-term viability of L-SPIO-loaded cells. HeLa cells were first seeded in a 24-well plate (5 × 10^4^/well) overnight and treated with 1 nmole/well of L-SPIOs for 12 h in 10% FBS-DMEM. After different time periods of culture, cells were washed, trypsinized and counted with a hemocytometer. (C) Magnetic properties of L-SPIO-loaded cells. HeLa and PC-3 cells were treated with the above L-SPIOs for 12 h in 10% FBS-DMEM and harvested at different time points. L-SPIO-positive cells were separated by a magnet stand and counted with a hemocytometer. All experiments were performed in triplicate. Results are mean ± SEM (n = 3).

After lipid coating, L-SPIOs retained the magnetic properties of SPIOs indicated on the magnet stand (Fig. 2A, left). When cationic surface-charged L-SPIOs were introduced into HeLa cells with the assistance of magnetic field gradient for 12 h, the cells demonstrated similar magnetic properties as L-SPIOs on magnet stand (Fig. 2A, right). Cell proliferation assay was used to determine whether L-SPIOs affected cell viability. After seeding cells in culture plates, the cell number of control and L-SPIO-loaded cells were counted using the hemocytometer in different day. As shown in Figure 2B, there was no effect of L-SPIO loading on cell growth up to 11 days. HeLa and PC-3 cells were loaded with L-SPIOs to further assess the duration of magnetic property of the L-SPIO-loaded cells (Fig. 2C). After loading L-SPIOs at different time points, the SPIO-positive cells were separated by a magnet stand and counted with a hemocytometer. More than 80% of the SPIO-loaded cells still preserved the magnetic property when cultured for 7 days (Fig. 2C).

### Effects of L-SPIO-loading on neuronal cell differentiation

**Figure 3:**
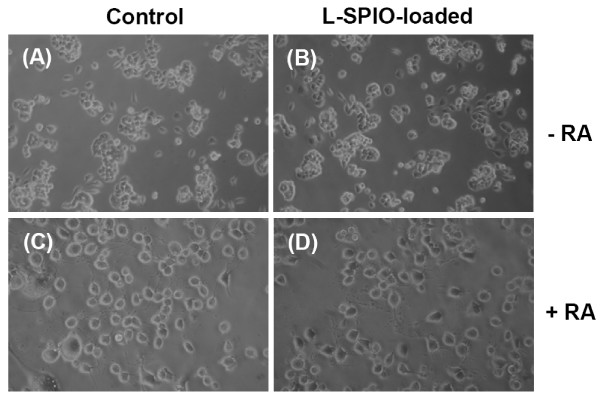
**Differentiation status of Neuro-2a**. Neuro-2a cells were treated with or without L-SPIOs (4 nmol/well) for 24 h in 10% FBS-DMEM. After L-SPIO-loading, Neuro-2a cells were then washed and exposed to 5 μM of retinoic acid for differentiation indicated by neurites outgrowth. Phase Images of Neuro-2a cells before (A, B; 100× magnification) and after (C, D; 200× magnification) retinoic acid (RA) treatment in control or L-SPIO-loaded cells as indicated.

We next evaluated the biocompatibility by testing the effect of L-SPIO-loading on neuronal cell differentiation. To this end, Neuro-2a cells, an albino mouse neuroblastoma cell, were treated with L-SPIOs (4 nmole lipid/well, equivalent to 7.5 μg SPIO per well) for 24 h. Following the SPIO-loading, Neuro-2a cells were treated with 5 μM of retinoic acid for another 60 h to induce neuronal differentiation. Figure 3 showed that neuronal differentiation was evident in both control and L-SPIO-loaded Neuro-2a cells with no significant difference in morphological differentiation between control and L-SPIO- loaded Neuro-2a cells (control: 86.5 ± 4.3%; L-SPIO-loaded: 82.7 ± 7.4%).

### In vivo study of tumor cell growth by optical and MR imaging

**Figure 4:**
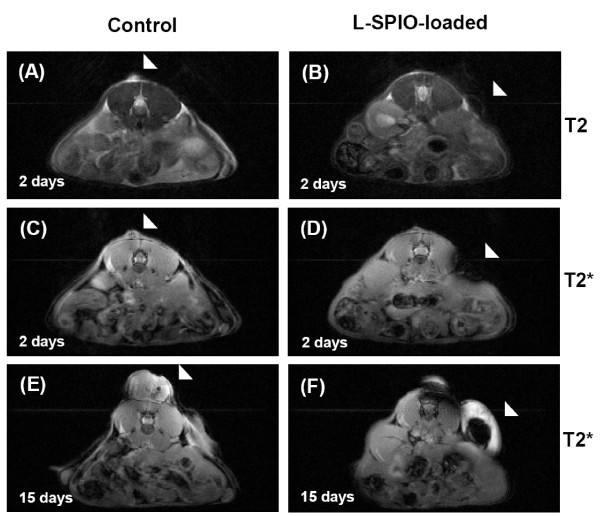
**MR imaging of L-SPIO-loaded cancer cells**. L-SPIO loaded CT-26 cells were injected into the dermis of Balb/c mice. MRI imaging was performed in these mice bearing orthotopic colorectal tumor (triangle) 2 days (A~D) and 15 days (E, F) after subcutaneous injection of control and L-SPIO-loaded CT-26 cells. *T2*-weighted images of tumor nodule from CT-26 cells without (A) and with L-SPIOs (B) were shown. T2*-weighted images of tumor nodule from CT-26 cells without (C, E) and with L-SPIOs (D, F) were shown.

**Figure 5:**
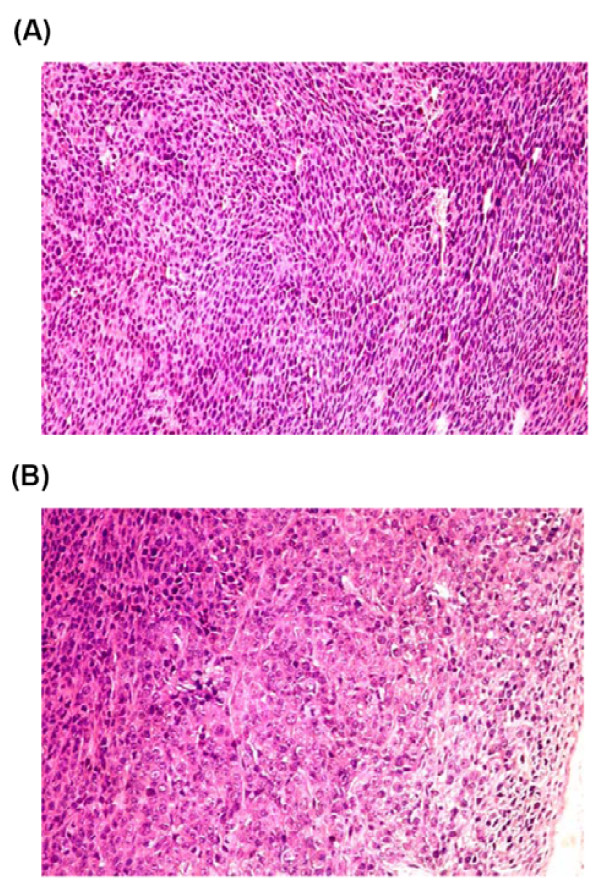
**Histological examination of subcutaneous tumors in Balb/c mice**. L-SPIO loaded CT-26 cells were injected into the dermis of Balb/c mice to form tumors. Sections of the subcutaneous tumor growing from inoculated L-SPIO-loaded CT-26 cells are indicated. Histochemical H/E staining showed proliferation cells at central (A) and peripheral regions (B) of the tumor nodule growing from L-SPIO-loaded CT-26 cells. (200× magnification).

To further know whether the SPIO-loaded cells were suitable for in vivo study, control or SPIO-loaded CT-26 cells (3 × 10^6^) were injected subcutaneously into the back of Balb/c mice followed by MR imaging at Day 2 and Day 15 after tumor cell inoculation by a clinical 3T MR system. The *T2*-weighted and T2*-weighted MR images were obtained as described in Methods (Fig. 4). In control cells (images A, C and E), tumor proliferation was obvious in both *T2*-weighted (image A) and T2*-weighted (image C) MR images at Day 2, and tumor size increased remarkably to 5 mm in diameter at Day 15 (image E). When the Balb/c mice were injected with L-SPIO-loaded cells, the *T2*-weighted (image B) and T2*-weighted (image D) images both revealed cell growth at Day 2. Tumor grew to a comparable size as compared to control cell inoculation at Day 15. In order to determine cell viability of the central region (2.5 mm in diameter) of tumor, which are positive in MRI signal as indicated by arrow in Fig. 4F, histological staining and examination were employed. Tissue sections showed that the subcutaneous tumor was a poorly differentiated carcinoma, consisting of mostly viable tumor in both the center and peripheral regions of the tumor (Fig. 5). The histological examination also showed no difference between tumors formed by control and L-SPIO-loaded CT-26 cancer cells (data not shown). These results demonstrated that CT-26 cells loaded with L-SPIOs retained the magnetic property for *in vivo *MR studies up to 15 days. The tumor growth was not affected by L-SPIOs and the MR images were readily distinguishable from those of control cells, thus allowing for a long-term tracking.

## Discussion

Nanotechnology provides size-controlled nanostructures such as quantum dots and magnetic iron oxide nanoparticles for molecular imaging [21]. For cellular MR imaging, nonspecific labeling of primary cells and stem cells with these nanoparticles has been used in biomedical research. From technical aspect, loading iron oxide nanoparticles with transfection reagents such as liposomes, polylysine and protamine sulfate from the market is achievable [10-14]. But the sizes of these complexes in the present of serum are greater than 150 nm or even up to 1 μm in diameter, thus limiting their use in targeted delivery and live cell imaging [22-25]. In this study, we have developed a novel formulation of functionalized SPIOs by encapsulating of SPIOs into diacyllipid formed- micelles composing of DOTAP and PEG-DSPE. This strategy resulted in small and stable cationic nanoparticles with magnetic iron oxide in the core, allowing an efficient cellular loading with low cytotoxicity for *in vivo *imaging.

PEG-lipid, due to its high water solubility and low toxicity, is self-assembled into nanosized micelles for drug delivery [16]. But, the long chain of PEG blocks lower the uptake of micelles by the reticuloendothelial system, thereby resulting in the long circulation times in blood [26]. In this regard, cationic lipids also increase solubility of the nanoparticles in the physiological buffers while is capable of loading nanoparticles into cells, presumably via the negatively charged biomolecules on cell membrane. With low transition temperature and low toxicity, DOTAP has been demonstrated to be an effective cationic lipid for transfection [27-29]. In our new formulation, we circumvent the disadvantage of PEG in prohibiting particle cellular uptake by including DOTAP to overcome the PEG shielding effect, thus facilitating nanoparticle translocation across cell membrane.

Another interesting feature in our formulation is that application of magnetic field in nanoparticle-loading resulted in significant increase of cellular uptake in a dose- and time-dependent manner (Fig 1). Of note, when initially loaded with high amount of magnetic nanoparticles, more than 80% of HeLa or PC-3 cells could maintain magnetic property up to 7 days, indicating the long retention of iron oxide (Fig 2). The loaded nanoparticles were predominately located in the cytoplasm rather than on the cell membrane of these cells (Fig 1D). In our previous experiments using lipid-enclosed quantum dots (LEQD) [30], the durations of internalized LEQD could be detected by third harmonic generation (THG) microscopy in these loaded cells for more than two weeks. Since only lipid-coated quantum dots but not bare quantum dots could generate strong THG signal, we inferred here that the SPIOs inside cells may be still in lipid-coated form within time of these experiments. Further, it should be mentioned that magnet-assisted gene delivery has been shown to increase the efficiency of pDNA transfection but with a significant increase of cellular toxicity in human umbilical vein endothelial cell (HUVEC) [31]. In addition, long term expose of anionic iron oxide (Fe_2_O_3_) nanoparticles, at concentration range from 150 μM to 15 mM, caused PC12M cytotoxicity in both cell growth and neurites outgrowth [32]. Given the negligible cytotoxicity of L-SPIOs loading at high concentration both in cancer cell growth (HeLa, PC-3) and neural cell differentiation (Neuro 2a) (Fig 2B and 3), L-SPIOs thus have a potential in efficient drug delivery with low cytotoxicity and long duration.

Iron oxide nanoparticles were widely used in labeling cells due to their ability to create apparent image upon cellular internalization and particle clustering [33]. In vitro labeling of lymphocytes, such as T-cells and dendritic cells, and their tracking in vivo after injection into mice can be also visualized their pathogenesis and evolution using clinical MRI [34]. It also demonstrated that implantation of iron-oxide-labeled transplanted cells can be monitored within individual animals for a prolonged time by MR imaging [6,35]. Therefore, it is feasible to monitor the prelabeled stem cells with iron oxide in differentiation and visualize the presence and migration of transplanted stem cells to the cerebral ischemia [36,37]. Nevertheless, it was believed that the intracellular concentration of SPIOs decreased when cell proliferated, hence reducing the MRI signals for long-term in vivo tracking. However, results from our in vivo study of tumor growth (Fig 4) clearly demonstrated that even after 15 days the MRI signals were still detectable. Histological data showed that the tumor mass formed from injected L-SPIO-loaded tumor cells were viable both the center and peripheral regions (Fig. 5), with similar growth potential as compared to non-SPIO-loaded cells (Fig. 4E, 4F). This is consistent with a recent report by Nelson *et.al*. showing that a long duration imaging of SPIO-loaded cells in SCID mice [38]. Thus, the biocompatibility nature of L-SPIO nanoparticles supports their use for long-term cell tracking and imaging in the in vivo system.

## Conclusion

We have developed a novel cationic lipid-based nanoparticle of SPIOs with high loading efficiency, low cytotoxicity and long-term imaging signals. The potential of our lipid-coated magnetic nanoparticles is at least three folds. First, the iron oxide moiety helps the magnet-enhanced nanoparticle delivery and MR imaging, as well as for potential hyperthermia therapy. Second, targeted properties can be achieved by adding targeted ligands onto PEG linker of the lipid shell. Third, it provides size-controlled nanostructures that integrate biomolecules onto their lipid coat for efficient drug delivery. We believe that this multifunctional nanoparticle will provide an efficient way for in-vivo cell tracking [30], and therefore make a potential contribution to nanomedicine research in the future.

## Abbreviations

SPIOs: Superparamagnetic Iron Oxide Nanoparticles; MR: Magnetic Resonance; L-SPIOs: Lipid-coated SPIOs; DOTAP: 1,2-dioleoyl-3-(trimethylammonium) propane; PEG-DSPE: Polyethylene-glycol-2000-1,2-distearyl-3-sn-phosphatidylethanolamine; Rodamine-DOPE: Rhodamine-dioleoyl-phosphatidylethanolamine; DMEM: Dulbecco's Modified Eagle's Medium; DLS: Dynamic Light Scattering; LDV: Laser Doppler Velocimetry.

## Competing interests

The authors declare that they have no competing interests.

## Authors' contributions

HCH, CYM and FHC conceived and designed the experiments. HCH, KC, CYC, CWL and JHC performed the experiments. HCH, PYC, CWL, JHC, ZFC and FHC analyzed the data. HCH, PYC, ZFC and FHC wrote the paper. All authors have read and approved the final manuscript.
